# Signaling Pathways Involved in the Regulation of mRNA Translation

**DOI:** 10.1128/MCB.00070-18

**Published:** 2018-05-29

**Authors:** Philippe P. Roux, Ivan Topisirovic

**Affiliations:** a Institute for Research in Immunology and Cancer, Université de Montréal, Montréal, Québec, Canada; b Department of Pathology and Cell Biology, Faculty of Medicine, Université de Montréal, Montréal, Québec, Canada; c Lady Davis Institute for Medical Research, Jewish General Hospital, Montréal, Québec, Canada; d Gerald Bronfman Department of Oncology and Departments of Experimental Medicine and Biochemistry, McGill University, Montréal, Québec, Canada

**Keywords:** mRNA translation, mTOR, MAPK, MNK, RSK, eIF4E, signal transduction, mRNA, mitogen-activated protein kinases, protein phosphorylation, translational control

## Abstract

Translation is a key step in the regulation of gene expression and one of the most energy-consuming processes in the cell. In response to various stimuli, multiple signaling pathways converge on the translational machinery to regulate its function. To date, the roles of phosphoinositide 3-kinase (PI3K)/AKT and the mitogen-activated protein kinase (MAPK) pathways in the regulation of translation are among the best understood. Both pathways engage the mechanistic target of rapamycin (mTOR) to regulate a variety of components of the translational machinery. While these pathways regulate protein synthesis in homeostasis, their dysregulation results in aberrant translation leading to human diseases, including diabetes, neurological disorders, and cancer. Here we review the roles of the PI3K/AKT and MAPK pathways in the regulation of mRNA translation. We also highlight additional signaling mechanisms that have recently emerged as regulators of the translational apparatus.

## INTRODUCTION

Steady-state mRNA levels do not correlate well with the protein composition in the cell (1, 2), suggesting that posttranscriptional mechanisms of regulation of gene expression play a major role in shaping proteomes. Translation is a key step in the regulation of gene expression (reviewed in references 3 and 4) and is energy costly (5, 6). As such, translation is tightly controlled by signaling pathways that sense various stimuli, including environmental stresses (e.g., heat shock or UV irradiation), extracellular stimuli (e.g., hormones or growth factors), and intracellular cues (e.g., nutrients, energy status, or amino acids) (4, 7, 8). Here we summarize the current knowledge on major signaling pathways involved in translational control via phosphorylation of the components of the translational machinery. In particular, we focus on the mammalian/mechanistic target of rapamycin (mTOR) and the mitogen-activated protein kinases (MAPK). Another important pathway in translational control involves eukaryotic initiation factor 2 (eIF2) kinases and phosphatases and has been reviewed elsewhere (9, 10). Components of the translational machinery actively participate in the regulation of protein synthesis in homeostasis and when dysregulated are thought to contribute to pathological conditions, including cancer. Hence, the regulation and functional consequences of phosphorylation of the components of the translational machinery are described in detail below.

## mTOR

**FIG 1:**
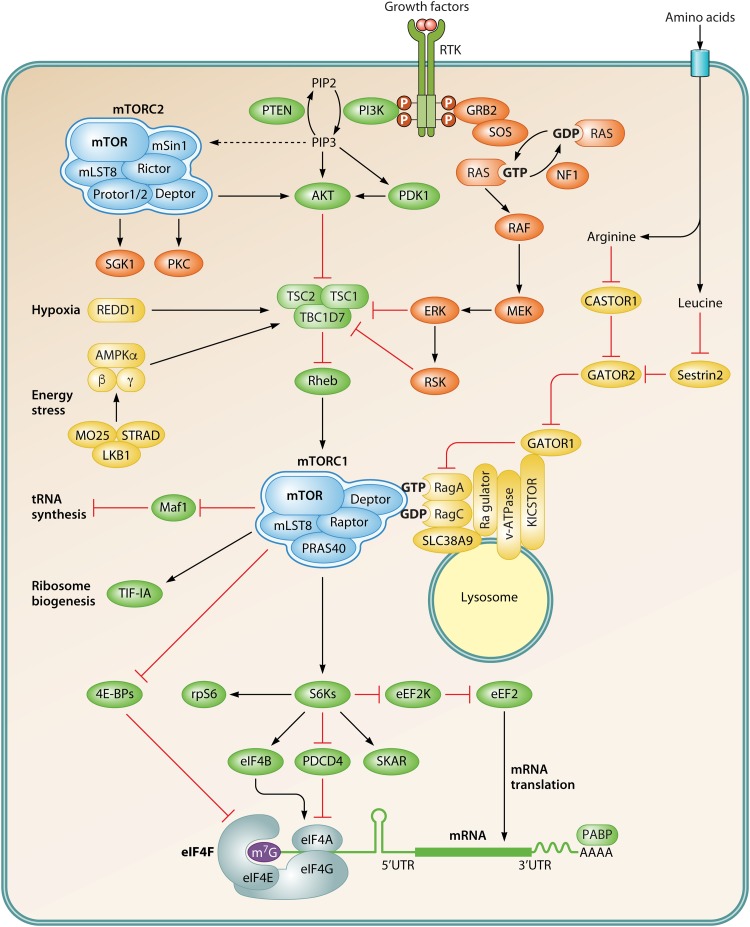
Schematic representation of mTOR signaling to the translational machinery. Growth factors stimulate mTORC1 signaling by activating receptor tyrosine kinases (RTKs) located at the plasma membrane. Various adaptor proteins convert these extracellular signals by stimulating the PI3K/AKT and Ras/ERK pathways. Many additional cues promote mTORC1 activation, including glucose and amino acids via small Rag GTPases, which help translocate mTORC1 to the surface of lysosomes. In turn, insufficient energy resources (energy stress) and hypoxia inactivate mTORC1 via the LKB1/AMPK pathway and REDD1, respectively. mTORC2 also responds to agonists that stimulate the production of phosphatidylinositol-3,4,5-triphosphate (PIP3) and promotes the activity of AGC kinase family members (PKC, AKT, and SGK) by phosphorylating residues located in their hydrophobic motifs. mTORC1 modulates mRNA translation by promoting the phosphorylation of downstream substrates, including the 4E-BPs and S6Ks, the latter having phosphorylation substrates of their own (e.g. eIF4B, rpS6, PDCD4, and SKAR). Red T-bars represent inhibitory signals, whereas black arrows indicate stimulatory signals. P denotes phosphorylation. Abbreviations and detailed explanations about this signaling network are provided in the text.

mTOR is a conserved Ser/Thr kinase that integrates stimuli including growth factors, hormones, cellular energy status, and nutrient and oxygen availability (Fig. 1) to adjust proliferation (increase in cell number) and growth (increase in cell volume/mass) (11). mTOR stimulates anabolic processes, such as protein and lipid synthesis, and it is found in two structurally and functionally different complexes: mTOR complexes 1 and 2 (mTORC1 and -2) (12). mTORC1 is composed of mTOR, the scaffolding protein raptor (regulatory-associated protein of TOR), the GTPase β-subunit like protein (GβL) (also known as mLST8), and deptor (disheveled, Egl-10, pleckstrin [DEP] domain-containing mTOR-interacting protein) (13, 14). Whereas mLST8 and deptor are found in both mTORC1 and mTORC2 (13, 14), rictor (rapamycin-insensitive companion of TOR), mSIN1 (mammalian stress-activated protein kinase [SAPK]-interacting protein), and protor (proline-rich protein 5, also known as PRR5) are specific components of mTORC2 (15–19). In addition to differences in their composition, mTORC1 and mTORC2 govern distinct cellular processes via phosphorylation of largely nonoverlapping substrates. Several mTORC1 substrates are involved in the regulation of mRNA translation, including the eukaryotic translation initiation factor 4E (eIF4E)-binding proteins (4E-BPs), 70-kDa ribosomal S6 kinases (S6Ks) 1 and 2 (reviewed in references 20, 21, and 22), and LARP1 (La ribonucleoprotein domain family member 1) (23–25). With respect to protein synthesis, mTORC2 associates with ribosomes (26, 27), and it was found to promote cotranslational phosphorylation and folding of nascent AKT polypeptides (26).

In addition to functional and structural distinctions, mTORC1 and mTORC2 are differentially sensitive to rapamycin, which is a naturally occurring allosteric inhibitor of mTOR (28–30) (Table 1). Together with the immunophilin FKBP12 (FK506-binding protein of 12 kDa), rapamycin associates with the FRB (FKBP12-rapamycin-binding) domain of mTOR (31). These studies led to the alternative conclusion that the rapamycin-FKBP12 complex prevents binding of mTORC1 to its substrates by steric hindrance via reduction in the size of the active-site cleft of mTOR. This steric hindrance model is consistent with the differential sensitivity of mTORC1 substrates to rapamycin. For instance, under most conditions tested, rapamycin potently suppresses S6K phosphorylation whereas it has only a marginal effect on 4E-BP phosphorylation levels (32). These differences may lie in the intrinsic capacity of particular phosphorylation sites to serve as mTORC1 substrates, which was shown to determine their sensitivity to modulators of the pathway, such as rapamycin (24, 33). Unlike mTORC1, mTORC2 appears to be much less sensitive to rapamycin, at least under conditions wherein rapamycin has been applied for less than 12 h in cell culture. These findings paved the way for the identification of a second generation of mTOR inhibitors that target its catalytic site, irrespective of whether mTOR is found in mTORC1 or mTORC2 (28) (Table 1). Many of these compounds are currently being tested in clinical trials in oncology (34). Rapa-Link1 is a third-generation inhibitor that simultaneously acts as an allosteric inhibitor and targets the active site of mTOR (35). Notably, Rapa-Link1 is effective against tumor cells which harbor mTOR mutations that render them resistant to rapalogs and active-site mTOR inhibitors (35) and has shown promising results in preclinical cancer models (36).

**TABLE 1:** Small-molecule inhibitors of mRNA translation and upstream pathways^*a*^

Target	Inhibitor(s)	Mechanism of action
mTORC1	Rapamycin (sirolimus), everolimus (RAD001), temsirolimus (CCI-779), ridaforolimus (AP23573)	Rapamycin and the rapalogues bind to FKBP12, which allows the formation of a ternary complex with mTOR; the rapamycin-FKBP12 dimer binds mTOR outside its kinase domain, and it is believed that this interaction interferes with the binding of mTOR and its substrates
PI3K/mTOR	BEZ235 (dactolisib), PI-103, XL765 (voxtalisib), BGT226, PF-05212384 (gedatolisib)	ATP-competitive inhibitors of mTOR and PI3K (multiple isoforms)
mTORC1/2	MLN0128 (sapanisertib), AZD8055, Torin1, PP242 (torkinib), OSI-027, Rapa-Link1	ATP-competitive inhibitors of mTOR (inhibit either mTORC1 or mTORC2) (Rapa-Link1 simultaneously acts as ATP-competitive and allosteric inhibitor)
PI3K	BKM120 (buparlisib), GDC-0941 (pictilisib), BAY 80-6946 (copanlisib), ZSTK474, GDC-0032 (taselisib)	Pan-class I PI3K ATP-competitive inhibitors
	BYL719 (alpelisib), SAR260301, GS-1101 (idelalisib), INCB040093, AMG319, TGR-1202, IPI-145 (duvelisib), GSK2636771	Isoform-specific PI3K ATP-competitive inhibitors
AKT	MK2206	Pleckstrin homology domain-dependent allosteric inhibitor of AKT that promotes AKT relocalization to the cytoplasm and prevents its phosphorylation by PDK1 and mTORC2; MK2206 is more selective toward AKT1/2 than toward AKT3
	KRX-0401 (perifosine)	Alkyl-phosphocholine that targets cellular membranes and thereby inhibits AKT activation, as well as many other membrane-dependent events
	GSK690693	ATP-competitive inhibitor of Akt1/2/3
	GDC-0068 (ipatasertib)	Non-ATP-competitive inhibitor of Akt1/2/3
S6K1	PF-4708671	Inhibits S6K1-dependent phosphorylation of substrates; mechanism of action is unavailable
	LY2584702	ATP-competitive inhibitor of S6K1
MNK	eFT508	ATP-competitive inhibitor of MNK1/2
	BAY 1143269	MNK1 inhibitor with undisclosed mechanism of action
	Cercosporamide, CGP57380, CGP052088	Poorly selective MNK inhibitors that target the ATP-binding domain of MNK1/2
RSK	LJH685 (and related LJI308), SL0101, BI-D1870	ATP-competitive inhibitor of the RSK N-terminal kinase domain
	FMK	Covalent inhibitor of the C-terminal kinase domain of RSK1/2/4
eIF4E	LY2275796	Reduction of eIF4E expression using antisense oligonucleotide
	Cap analogues, including 4Ei-1	Inhibition of eIF4E binding to 5′ cap of mRNA
	4EGI-1, 4E1RCat, 4E2RCat	Inhibition of eIF4E-eIF4G interaction
eIF4A	Silvestrol, hippuristanol, pateamine A	Inhibition of eIF4A helicase activity

a This table includes selected small-molecule inhibitors that target components of the translation machinery (eIF4E and eIF4A) or upstream pathways involved in translational control (mTOR, PI3K, AKT, S6K, MNK, and RSK).

## REGULATION OF mTORC1 BY HORMONES AND GROWTH FACTORS

Hormones and growth factors, including insulin and insulin-like growth factor (IGF), stimulate receptor tyrosine kinases (RTKs), such as insulin receptor or IGF receptor, and activate phosphoinositide 3-kinase (PI3K) via associated adaptor molecules (e.g., IRS-1 and -2). PI3K generates phosphatidylinositol-3,4,5-trisphosphate (PIP_3_) from phosphatidylinositol 4,5-bisphosphate (PIP_2_) (reviewed in reference 37). Conversely, PIP_3_ is hydrolyzed to PIP_2_ by PTEN (phosphatase and tensin homologue), which thus acts as a negative regulator of PI3K (38). PIP_3_ recruits phosphoinositide-dependent kinase 1 (PDK1) and AKT to the plasma membrane (Fig. 1), where PDK1 activates AKT by phosphorylating a residue localized in its activation loop (Thr308 in human AKT1) (reviewed in reference 39). mTORC2 phosphorylates the hydrophobic motif of AKT (Ser473 in human AKT1) (40), which increases AKT activity toward a subset of substrates (16, 41). Tuberous sclerosis complex (TSC), a negative regulator of mTOR, consists of the TSC1 scaffolding protein and TSC2, which is a GTPase-activating protein (GAP) toward Rheb (Ras homologue enriched in brain) (42). TSC stimulates hydrolysis of Rheb-GTP to the inactive Rheb-GDP form (43, 44). In addition to TSC1 and TSC2, TBC1D7 (Tre2-Bub2-Cdc16-1 domain family member 7) acts as a third component of TSC (45). Rheb is a small GTPase that stimulates mTORC1 in its active, GTP-bound form (46). Rheb was shown to bind to mTOR and cause a global conformational change that allosterically promotes mTOR activation (47). It was thought that AKT phosphorylates TSC2 and inhibits its GAP activity, which results in increased Rheb-GTP levels and mTORC1 activation (43, 44, 48, 49). This model was, however, challenged by the observation that Rheb recruits TSC to the lysosomal surface, whereby TSC2 maintains Rheb in its inactive GDP-bound state (50). AKT-mediated phosphorylation of TSC2, which only marginally reduces its GAP activity, leads to dissociation of TSC from the lysosome, thereby allowing Rheb-GTP loading and mTORC1 activation (50).

PRAS40 is a negative regulator of mTORC1 (51–55). AKT phosphorylates PRAS40 (at Thr246 in humans) and stimulates its dissociation from mTORC1 (53, 54). mTORC1 phosphorylates multiple residues on PRAS40, which indicates that PRAS40 is also an mTORC1 substrate. PRAS40 contains a TOR signaling (TOS) motif that interacts with raptor and thus may compete for raptor binding with other mTORC1 substrates (e.g., 4E-BPs and S6Ks) (56, 57).

In addition to PI3K, growth factors (e.g., epidermal growth factor [EGF]) activate mTORC1 via the Ras GTPase (reviewed in reference 58). Oncogenic Ras signaling has been linked to elevated mTORC1 activity. Inactivating mutations in the *NF1* gene, whose protein product (neurofibromin) acts as a GAP and inactivates Ras, leads to mTORC1 hyperactivation (59, 60). Ras signals via the RAF/MEK/ERK axis to activate mTORC1, whereby extracellular signal-regulated kinase (ERK) phosphorylates TSC2 and raptor directly (61–64) or via the 90-kDa ribosomal S6 kinases (RSKs) (65–68).

## REGULATION OF mTORC1 BY NUTRIENTS AND METABOLITES

Amino acids stimulate mTORC1 (69, 70). In Saccharomyces cerevisiae, this is achieved via the Vam6/VPS39-Gtr1/Gtr2 axis. Vam6/VPS39 was shown to promote GTP loading onto the Gtr1 GTPase, which is a subunit of the vacuolar-membrane-associated EGO complex that associates with TORC1, resulting in its activation (71, 72). RagA/B and RagC/D are mammalian orthologues of Gtr1 and Gtr2, respectively (73, 74). RagA or RagB forms heterodimers with RagC or RagD, and their activity is controlled by the ragulator (75), which anchors Rags to the lysosome (76). Ragulator, or LAMTOR (late endosomal/lysosomal adaptor and MAPK and mTOR activator), is a pentameric complex consisting of p18, p14, MP1 (MEK binding partner 1), HBXIP (hepatitis B virus X-interacting protein), and C7orf59, which are also known as LAMTOR1 to -5. In addition to mTOR, LAMTOR2 (p14) and LAMTOR3 (MP1) are also implicated in MEK1 and ERK1/2 activation (77). In their active form, wherein RagA/B and RagC/D are GTP and GDP loaded, respectively, Rag GTPases recruit mTORC1 to the lysosomal surface, which facilitates mTORC1 activation by Rheb (78, 79). p62 interacts with raptor to stimulate mTORC1-Rag association on the lysosomes (80). GAP activity toward Rags 1 (GATOR1) is a heterotrimeric complex comprising of Nprl3, Nprl2, and DEPDC5, which acts as a RagA/B GAP and inhibits mTORC1 (81). GATOR2 inhibits GATOR1 via suppression of DEPDC5. Different amino acids activate mTORC1 via distinct pathways. SLC38A9, a lysosomal membrane-associated protein, and the cellular arginine sensor for mTORC1 complex 1/2 (CASTOR1/2) activate mTORC1 in response to arginine (82–84). The p53-inducible proteins sestrin1 and -2 mediate the effects of leucine on mTORC1 signaling (85). Akin to CASTOR1/2, sestrins bind to GATOR2 and suppress mTORC1 signaling. Addition of leucine leads to dissociation of sestrin-GATOR2 complexes, leading to mTORC1 activation (86). During amino acid starvation, GCN2 upregulates ATF4 and increases the expression of sestrin2, which correlates with mTORC1 inhibition (87). The transport of leucine, arginine, and glutamine into the cell also plays a role in mTORC1 regulation (88). For instance, cellular uptake of glutamine by ASCT2 and its subsequent rapid efflux by obligatory exchange with essential amino acids (e.g., leucine) by LAT1/2 transporters are required for mTORC1 activation (89).

mTOR activity is also controlled by metabolic intermediates. Isocitrate dehydrogenases 1 and 2 (IDH1/2) mutations in cancer (90) result in accumulation of d-2-hydroxyglutarate (d-2HG) (91). It was initially reported that d-2HG suppresses mTORC1 by binding to ATP5B and interfering with ATP production (92). mTOR, however, appears to be frequently activated in brain cancers harboring IDH1 or IDH2 mutations (91, 93). Consistent with this, d-2HG was recently shown to positively regulate mTOR (94, 95), in part by inhibiting KDM4A, an αKG-dependent enzyme of the Jumonji family of lysine demethylases (94). KDM4A negatively regulates mTORC1 and mTORC2 via associating with deptor (an inhibitor of mTORC1 and mTORC2) and precluding its targeting for degradation by SCF^βTrCP^ (94). Modulation of mTOR activity by intermediate metabolites suggests the presence of regulatory feedback loops whereby mTOR activity, and thus the protein synthesis rate, is adjusted to the flux of specific metabolic pathways to maintain energy homeostasis.

## REGULATION OF mTORC1 BY ENERGY STATUS AND OXYGEN AVAILABILITY

Alterations in cellular energetics are sensed by the AMP-activated protein kinase (AMPK) (96). AMPK is a heterotrimeric Ser/Thr kinase that contains a catalytic α and regulatory β/γ subunits (97, 98). Increased intracellular AMP/ATP and ADP/ATP ratios coincide with increased AMP or ADP binding to the AMPK γ subunit, which stimulates phosphorylation of the α subunit (on Thr172 in human protein) by serine/threonine kinase 11 (STK11/LKB1) (98). Glucose serves as a major energy source, and reductions in glucose flux are paralleled by reduced mTORC1 signaling (Fig. 1). Reduction in the glycolytic intermediate fructose-1,6-bisphosphate results in aldolase-mediated formation of a lysosomal-membrane associated complex, comprising AMPK, LKB1, axin, v-ATPase and ragulator, which leads to AMPK activation (99). AMPK conserves cellular energy by downregulating anabolic processes, including protein synthesis, which is mediated via mTORC1 inhibition (98, 100). AMPK phosphorylates and activates TSC2 (101) and phosphorylates and sequesters raptor in concert with 14-3-3 proteins (102).

Reduction in oxygen hinders ATP production, thereby activating AMPK (100). Hypoxia also inhibits mTORC1 via REDD1 (regulated in development and DNA damage response 1) (103). REDD1 downregulates mTORC1 by preventing the 14-3-3–TSC2 interaction and stabilizing TSC (104). Hypoxia-inducible proapoptotic protein BNIP3 (BCL2/adenovirus E1B 19-kDa-protein-interacting protein 3) also inhibits mTORC1 by directly associating with and inhibiting Rheb (105).

How mTORC1 activity is modulated by nutrients and/or alterations in cellular energetics to adjust protein synthesis rates *in vivo* is still poorly understood. Indeed, at the organismal level, nutrients regulate mTORC1 signaling in a fashion that appears to be significantly more multifarious than was previously expected (reviewed in reference 11).

## REGULATION OF mTOR BY PHOSPHORYLATION

Within its kinase domain, mTOR contains two phosphoacceptor sites (Ser2159 and Thr2164 in human mTOR) (106) that stimulate mTOR autophosphorylation (on Ser2481 in human protein) (107) and impact cell growth and proliferation (106). Phosphorylation of Ser2159 was recently shown to be mediated by the innate immune kinase TANK-binding kinase 1 (TBK1) (108), which activates mTORC1 in response to growth factors and innate immune agonists. In addition, phosphorylation of mTOR at the residue located in its HEAT (Huntington, elongation factor 3, PR65/A, TOR) repeat (Ser1261 in human protein) results in mTOR autophosphorylation and induction of cell growth (109). Raptor is also phosphorylated by mTOR on a number of residues (e.g., Ser863 in human protein), which upregulates mTORC1 activity (110). Some of the mTOR-dependent sites on raptor overlap those phosphorylated by ERK1/2 (61), suggesting that raptor is an important point of convergence for multiple signaling pathways.

## mTORC1 SIGNALING TO THE TRANSLATIONAL MACHINERY

**FIG 2:**
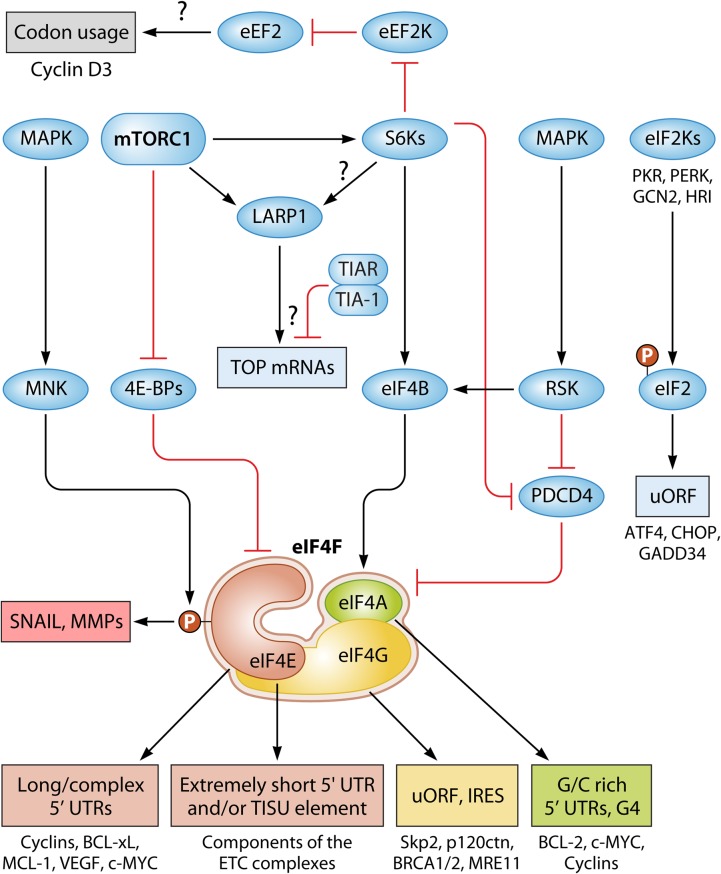
The mTOR and MAPK pathways affect the translatome by modulating the expression of specific subsets of mRNAs. Phosphorylation of the 4E-BPs by mTOR leads to their dissociation from eIF4E, which stimulates the interaction of eIF4E with eIF4G and assembly of the eIF4F complex. mTOR also promotes S6K-dependent phosphorylation of PDCD4 and eIF4B, which in turn regulate eIF4A levels and activity, respectively. eIF4E is the most limiting subunit of the eIF4F complex and is thus critical for the recruitment of eIF4A to the mRNA and unwinding of the secondary structure of its 5′UTR during ribosome scanning toward the initiation codon. The Ras/ERK pathway also regulates eIF4A activity by promoting RSK-dependent phosphorylation of eIF4B and PDCD4. eIF4E activity is also regulated by MAPK pathways by direct phosphorylation of eIF4E by the MNK protein kinases. Although the eIF4F complex regulates the translatome at a global scale, each subunit also appears to modulate the translation of specific subsets of transcripts. For instance, overexpression of eIF4E appears to selectively affect translation of mRNAs encoding proteins involved in tumor initiation and maintenance (e.g., cyclins, vascular endothelial growth factor [VEGF], and BCL-xL). Phosphorylation of eIF4E also seems to bolster the translation of mRNAs encoding proteins involved in tumor dissemination (e.g., SNAIL and MMP3). Various stresses activate eIF2 kinases (PERK, PKR, GCN2, and HRI) that phosphorylate eIF2 (alpha subunit), which reduces global protein synthesis but promotes the translation of mRNAs containing upstream open reading frames (uORFs), such as those encoding ATF4, CHOP, and GADD34. eIF4A promotes the translation of mRNAs with G/C-rich 5′ UTR sequences, such as the 12-nucleotide guanine quartet (CGG)_4_ motif, which can form RNA G-quadruplex structures. Red T-bars represent inhibitory signals, whereas black arrows indicate stimulatory signals. P denotes phosphorylation. Abbreviations and detailed explanations about this signaling network are provided in the text.

The most extensively studied mediators of the effects of mTORC1 on translation are 4E-BPs and S6Ks (29) (Table 2). More recently, La-related protein 1 (LARP1) emerged as a likely mediator of the effects of mTORC1 on translation of 5′-terminal oligopyrimidine tract (TOP) mRNAs (111, 112) (Fig. 2).

**TABLE 2:** Phosphorylation sites in human translation factors and associated proteins, regulatory kinases, and functional consequences of the phosphorylation^*a*^

Protein	Phosphorylation site(s)^*b*^ (reference) [main kinase(s)]	Biological function(s) (reference[s])
4E-BP1	Thr37 (317), Thr46 (317) [mTORC1 (117), GSK3β (126)]	Priming sites (118, 119, 116)
	Ser65 (317) [mTORC1 (117)], Thr70 (317) [mTORC1?/CDK1?]	Dissociation from eIF4E (118, 119, 116)
	Ser83 (129) [CDK1?], Ser85 (318) [?], Ser94 (319) [ATM?], Ser101 (320) [?], Ser112 (321) [CK2A1 (320)]	Unknown
4E-BP2	Thr37 (322), Thr46 (322) [mTORC1]	Priming sites (by analogy with 4E-BP1)
	Ser65 [mTORC1], Thr70 [mTORC1?/CDK1?]	Dissociation from eIF4E (by analogy with 4E-BP1)
eIF4E	Ser209 (243) [MNK1/2 (245)]	Unknown (244, 245), increases oncogenic activity and promotes translation of a subset of mRNAs (e.g., Mcl-1, MMPs, CCLs) (252)
eIF4GI	Ser1185 [PKCα (323), TBK1? (324)]	Modulates MNK binding (323)
	Ser1106, Ser1147, Ser1194 [mTORC1] (184)	Stimulation of translation of mRNAs containing uORFs (227) (?)
	Ser896 [Pak2] (310)	Inhibition of cap-dependent translation (310)
	Ser1231 (325) [CDK1?]	Inhibition of eIF4A/mRNA binding? (325)
eIF2α	Ser52 (326) [HRI, PKR, GCN2, PERK (reviewed in reference 4)]	Stabilizes the eIF2/GDP/eIF2B complex, thus preventing recycling of eIF2 (reviewed in reference 4)
eIF2β	S2, S67 [CK2 (327), mTORC1? (232)]	Stimulates translation and proliferation (327); stimulates eIF2α dephosphorylation (232)
rpS6	Ser235 (328) and Ser236 [S6K1/2, RSK (147)], Ser240, Ser244, and Ser247 [S6K1/2]	Unknown (329, 330, 122, 147), global translation rates increased in MEFs expressing a nonphosphorylatable form of rpS6 (148)
PDCD4	Ser67 [S6K1/2, AKT (163, 165)], Ser71 [?], Ser76 [RSK (331, 164)], Ser94 [?], Ser457 [S6K1/2, AKT (163, 165), RSK (164), PLK1? (332)]	Degradation by the ubiquitin-proteasome system and subsequent activation of eIF4A (163, 164, 165)
eIF4B	Ser406 (172) [?], Ser422 [S6K1/2 (170), AKT (172), RSK (171)], Ser422 [MELK? (333)]	Increases binding to eIF3 (173, 171), affects translation and proliferation (170)
eIF4H	Tyr12 (334), Tyr45 (334), Tyr101 (334), Ser193 (334) [?]	Unknown
eIF2Bε	Ser540 [GSK3] (335)	Inhibits recycling of eIF2 (335)
	Ser544 [DYRK] (336)	Priming site for GSK3 (336)
	Ser717/718 [CK2] (337)	Facilitates eIF2 binding (337)
eIF3	Subunit? [S6K1/2 (175)]	Paip1-eIF3 interaction (175)
	eIF3b: Ser83 (338), Ser85 (338), Ser125 (338) [?]	Unknown
	eIF3c: Ser39 (339), Ser166 (338), Thr524 (338), Ser909 (340) [?]	Unknown
	eIF3f: Ser46, Thr119 [CDK11] (341, 311)	Regulation of protein synthesis and apoptosis (341, 311)
	eIF3g: Thr41 (175), Ser42 (175) [?]	Unknown
	eIF3h: Ser183 (342) [?]	Increased oncogenic activity (342)
	eIF3i: Tyr445 (334) [?]	Unknown
eIF1	Tyr30 (334) [?]	Unknown
	Tyr72 (343) [?]	Stimulation of mRNA translation (343)
eIF5	Ser389, Ser390 [CK2] (344)	Promotes cell cycle progression (344)
eIF5B	Ser107 (338), Ser113 (338), S135 (338), S137 (340), S164 (338), S182 (338), S183 (340), S186 (338), S190 (338), S214 (345), S1168 (338) [?]	Unknown
eIF6	Ser174/175 [CK1] (346)	Nucleocytoplasmic shuttling (346)
	Ser235 [PKCβII] (347)	Dissociation of eIF6 from the 60S, 80S assembly (347)
eEF1A1	Thr432 [PKCδ] (348)	Activation (?) (348)
	Ser21 (349) [BRAF?]	Apoptosis (349)
	Ser300 [TβR-I] (350)	Inhibition of mRNA translation (350)
eEF1A2	Ser205, Ser358 [JNK (351)]	Degradation of newly synthesized polypeptides (351)
eEF2	Thr56 [eEF2K] (352)	Inhibits binding to the ribosome (353)
eEF2K	Ser78 (157) [mTOR?]	Inhibits CaM binding (157)
	Ser359 (155) [SAPK/p38δ?]	Inhibition (?) (155)
	Ser366 [S6K1, RSK] (150)	Inhibition (150)
	Ser398 [AMPK] (354)	Activation (354)
	Ser500 [PKA] (355)	Induces Ca^2+^-independent activity (355)

a This table includes selected phosphoacceptor sites identified in large-scale mass spectrometry-based experiments which await functional characterization (e.g., eIF5B; unknown kinase/function is indicated by a question mark), as well as phosphorylation sites with established role in translational control (e.g., 4E-BPs and eIF2α**)**. Further information on the as-of-yet functionally noncharacterized phosphorylated residues of the components of the translational apparatus can be found in the PhosphoSitePlus (www.phosphosite.org) or UniProt (www.uniprot.org) database. In the case of eIF4G1, the phosphorylation sites indicated are corrected from the published article (Ser1108, Ser1148, and Ser1192). Abbreviations: CDK, cyclin-dependent kinase; PKC, protein kinase C; Pak2, p21-activated kinase 2; HRI, heme-regulated eIF2α kinase; PKR, double-stranded-RNA-activated eIF2α kinase; GCN2, general control nonrepressed eIF2α kinase; PERK, double-stranded-RNA-activated protein kinase-like ER kinase; DYRK, dual-specificity tyrosine phosphorylation-regulated kinase; CK2, protein kinase CK2 (formerly known as casein kinase II); TβR-I, transforming growth factor β1 (TGF-β1) receptor; eEF2K, eukaryotic translation elongation factor 2 kinase; PKA, protein kinase A; SAPK, stress-activated protein kinase; TBK1, TANK-binding kinase 1, PLK1, polo-like kinase 1, MELK, maternal embryonic leucine zipper kinase; Paip1, polyadenylate-binding protein-interacting protein 1. Additional abbreviations are provided in the text.

b Amino acid numbering is based on human proteins.

### 4E-BPS.

Most cellular mRNAs are recruited to the ribosome via the 5′-mRNA cap structure (12). eIF4F is a heterotrimeric complex composed of eIF4E, eIF4G, and eIF4A (113). eIF4E acts as the cap-binding subunit, whereas eIF4A is an ATP-dependent DEAD box RNA helicase (12). eIF4G is a scaffold that bridges eIF4E-eIF4A interaction and that also associates with additional translation factors, including poly(A)-binding protein (PABP) and eIF3 (12, 114, 115). eIF4F recruits mRNA to the ribosome via interactions between eIF4E and the cap as well as eIF4G and eIF3 (12). 4E-BPs (4E-BP1, -2, and -3 in mammals) are repressors of eIF4F complex assembly (4). In their nonphosphorylated forms, 4E-BPs interfere with eIF4F complex assembly by binding to the site on eIF4E that overlaps with the eIF4G-binding motif, which blocks eIF4E-eIF4G association (116). mTORC1 activation results in hierarchical phosphorylation of 4E-BPs, whereby in human 4E-BP1, phosphorylation of Thr37 and Thr46 precedes phosphorylation of Thr70 and Ser65 (117–119). Phosphorylation of 4E-BPs facilitates their dissociation from eIF4E, which allows eIF4E-eIF4G interaction and eIF4F complex assembly (116, 118, 119) (Fig. 2). 4E-BPs are recruited to mTORC1 via raptor, which is mediated by the C-terminal TOS motif (FEMDI) (57). In mammals, 4E-BPs appear to chiefly mediate the effects of mTORC1 on proliferation, while S6Ks act as major effectors of mTORC1 on cell size (120–122). In contrast, in phylogenetically lower species, including Drosophila melanogaster, which express a single 4E-BP protein (d4E-BP), 4E-BP regulates both proliferation and cell size (123).

Although mTORC1 acts as a major 4E-BP kinase, additional kinases may also be involved (reviewed in reference 124). For instance, Ser/Thr kinase Pim-2 phosphorylates 4E-BPs (including the mTORC1-sensitive Ser65 site) in a number of leukemia and lymphoma cells (125). In addition, glycogen synthase kinase 3β (GSK3β) phosphorylates 4E-BP1 at Thr37/Thr46 (126). Casein kinase 1ε (CK1ε) phosphorylates 4E-BP1 (residues Thr41 and Thr50 in humans), which appears to be required for the phosphorylation of mTORC1-regulated sites and coincides with 4E-BP1 dissociation from eIF4E (127). Finally, cyclin-dependent kinase 1 (CDK1) phosphorylates 4E-BP1 at Thr70 and Ser83 during mitosis (128, 129). The mechanisms governing these alternative pathways of 4E-BP phosphorylation are not well established, and little is known regarding their physiological relevance.

### S6Ks.

In addition to the 4E-BPs, S6Ks also mediate effects of mTOR on protein synthesis (7, 22, 29, 130) (Fig. 2). There are two S6Ks in mammals (S6K1 and S6K2 [also referred to as S6Kα and S6Kβ]) (131). Notwithstanding that two separate genes (*RPS6KB1* and *RPS6KB2*) encode S6K1 and S6K2, the enzymes are highly homologous (reviewed in reference 132). Both mammalian S6Ks exist in distinct isoforms (p70- and p85-S6K1 and p54- and p56-S6K2), which are produced by alternative selection of translational start sites (133, 134). A third isoform of S6K1 (p31-S6K1) has also been described, which is generated via alternative splicing and results in a truncated kinase domain (135). p31-S6K1 plays an important role in cancer (136, 137). S6Ks have an evolutionarily conserved role in the regulation of cellular and organismal size. In mammals, S6Ks act as major effectors of mTORC1 on cell growth (138), whereas their effect on proliferation appears to be less pronounced (120–122). S6K1/S6K2 knockout mice are ∼15 to 20% smaller than their wild-type counterparts and suffer from perinatal lethality (122), which is consistent with the increased death of flies at the larval stage upon dS6K ablation (139). Size reduction is observed in S6K1 knockout mice (140) but not S6K2 knockout mice, which exhibit a modest increase in size (122). A similar reduction in cell size was observed in Drosophila upon ablation of its single S6K isoform (139). These findings suggest that S6K1 and S6K2 may play some nonoverlapping roles. For instance, S6K2, but not S6K1, has been implicated in the regulation of cell proliferation in cancer (141). S6K1 and S6K2 also play distinct roles in microRNA (miRNA) biogenesis (142). Finally, protein kinase C (PKC) has been shown to phosphorylate S6K2 (on S486 in humans) but not S6K1 (143).

The first step in activation of S6Ks is phosphorylation of several residues located in the C-terminal pseudosubstrate domain (144, 145). This is followed by phosphorylation of Thr residues within their activation loop (Thr229 in human p70-S6K1) and hydrophobic motif (Thr389 in human p70-S6K1) by PDK1 and mTORC1, respectively (reviewed in references 132 and 131). S6Ks are recruited to mTORC1 by raptor via their TOS motif (FDIDL in human S6Ks) (56, 57). In addition, GSK3 also phosphorylates S6Ks in their turn motif (Ser371 in human S6K1), which is thought to contribute to S6K activation (146).

**FIG 3:**
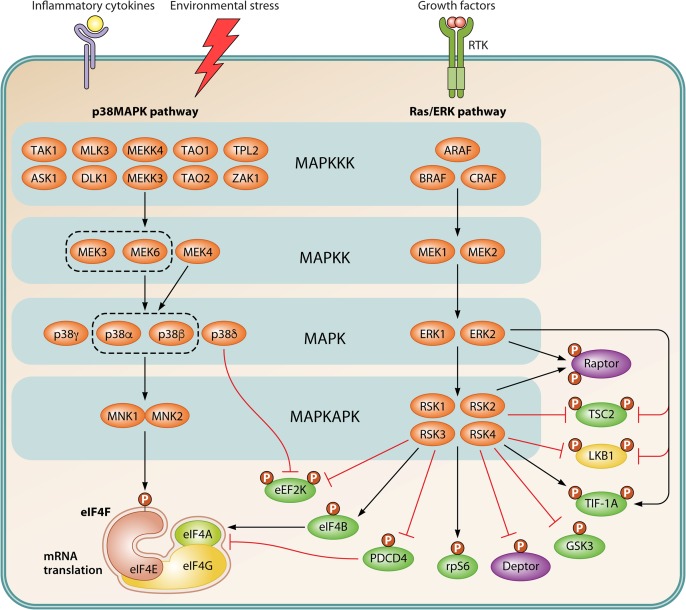
Schematic representation of MAPK signaling to the translational machinery. The Ras/ERK and p38MAPK pathways are activated by a wide range of stimuli, including cytokines, growth factors, and diverse environmental stresses. While many stimuli activate both MAPK pathways, stress stimuli and growth factors typically activate the p38MAPK and Ras/ERK signaling, respectively. While Ras/ERK signaling stimulates the activity of both RSK and MNK, the latter is also responsive to agonists of the p38MAPK pathway. MNK interacts with eIF4G and phosphorylates eIF4E on Ser209, a site that increases its oncogenic potential and facilitates the translation of specific mRNAs. Following activation of the Ras/ERK pathway, RSK phosphorylates rpS6, eIF4B, PDCD4, and eEF2K, which are important regulators of translation. RSK also modulates mTORC1 signaling by phosphorylating TSC2 and deptor. ERK and RSK regulate LKB1-dependent and -independent phosphorylation of raptor, resulting in increased mTORC1 signaling. ERK and RSK also collaborate in the regulation of ribosome biogenesis by promoting TIF-1A phosphorylation. Red T-bars represent inhibitory signals, whereas black arrows indicate stimulatory signals. P denotes phosphorylation. Abbreviations and detailed explanations about this signaling network are provided in the text.

The S6Ks regulate the phosphorylation of multiple components of the translational machinery (Fig. 1 and 2). S6Ks phosphorylate five residues in the C terminus of rpS6 (Ser235, Ser236, Ser240, Ser244, and Ser247 in humans). In turn, RSKs phosphorylate only Ser235 and Ser236 (122, 147) (Fig. 2 and 3). Expression of a nonphosphorylatable rpS6 mutant mirrors growth defects observed in S6K1/2 knockout mice (148), thereby indicating that the phosphorylation of rpS6 is involved in the regulation of cell growth. Expression of the nonphosphorylatable rpS6 mutant, however, moderately upregulates overall protein synthesis, whereas loss of S6Ks has only a marginal effect on global translation (122, 148). Finally, the S6K/rpS6 axis has been implicated in ribosome biogenesis (149).

S6Ks also phosphorylate eukaryotic elongation factor 2 (eEF2) kinase (eEF2K) (Ser366 in humans) (150) (Fig. 2). eEF2K belongs to a small group of proteins containing an α-kinase catalytic domain, and it functions as a negative regulator of protein synthesis through its ability to phosphorylate and inhibit eEF2 (151). eEF2K phosphorylates eEF2 (Thr56 in humans), which is a GTPase that promotes translocation of peptidyl-tRNA from the A site to the P site of the ribosome (152, 153). eEF2K is inactivated by insulin and other growth factors, which increase eEF2 function and elongation rates (150). This is mediated by the mTORC1/S6K axis, which phosphorylates eEF2K (at Ser366 in humans). While this site is also phosphorylated by RSK (150), ERK1/2 phosphorylates eEF2K at Ser359 (in humans) (154) and inhibits its function (155) (Fig. 3). Moreover, mTORC1 has been suggested to interfere with calmodulin-eEF2K binding by directly phosphorylating eEF2K (at Ser78 and Ser396 in humans) (150, 154). AMPK activates eEF2K via mTORC1 inhibition (156) and direct phosphorylation (at Ser398 in humans) (157). Understanding of the functional consequences of eEF2K phosphorylation is still incomplete. For instance, it has been shown that disruption of eEF2K mitigates the antineoplastic effects of mTORC1 inhibition, suggesting tumor-suppressive properties of eEF2K (158) (Fig. 2). Cell culture and xenograft experiments using a variety of cancer cell lines, however, point out that eEF2K may exhibit tumor-protective effects by decreasing energy consumption when nutrients are limiting (151, 159).

Programmed cell death 4 (PDCD4) is a proapoptotic factor that blocks eIF4G-eIF4A interaction (160, 161). This represses eIF4A activity and results in inhibition of cap-dependent translation (161, 162). S6Ks phosphorylate PDCD4 (on Ser67 and Ser457 in humans), which triggers its SCF^βTrCP^-dependent degradation (163). AKT and RSK can also target some of these PDCD4 sites (164, 165) (Fig. 3). eIF4B and eIF4H stimulate the RNA-unwinding activity of eIF4A by bolstering its processivity and establishing its directionality (116, 166–169). Several AGC kinases phosphorylate eIF4B on Ser406 (RSK and S6K) and Ser422 (S6K, AKT, and RSK), which appears to occur in a context-dependent manner (170–172) (Fig. 3). eIF4B stimulates cellular proliferation and survival, and its phosphorylation correlates with increased translation (171). It was also implied that eIF4B phosphorylation facilitates its association with eIF3, which was proposed to act as a scaffold for mTORC1 and S6K1 (173). S6Ks associate with eIF3 via the eIF3f subunit, whereby S6K-dependent phosphorylation of eIF3 increases eIF3-PABP-interacting protein 1 (Paip1) association, which correlates with upregulated translation (174, 175).

In addition, S6K1 has been suggested to stimulate translation of newly spliced mRNAs. According to this model, S6K1 is recruited to the exon-junction complex (EJC) by SKAR (S6K1 Aly/REF-like target) (176). Formation of S6K1-SKAR complexes with the EJC coincides with the phosphorylation of a number of mRNA-binding proteins, which is paralleled by increased translational activity of spliced mRNAs (177).

### LARP1.

La-related protein 1 (LARP1) is a conserved RNA-binding protein of the La motif (LAM)-containing factor family (111). LARP1 interacts with raptor and is phosphorylated by mTORC1, which is thought to modulate its mRNA-binding activity (23, 25, 178). While mTORC1 phosphorylates LARP1 on Ser689 and Thr692, other sites (Ser770 and Ser979) are phosphorylated by S6K1 and/or AKT (23), suggesting several layers of LARP1 phosphoregulation (Fig. 2).

LARP1 has been proposed to play a role in modulating stability and/or translation of TOP mRNAs (25, 178, 179) (Fig. 2). The interaction with TOP mRNAs requires the LARP1 family-specific DM15 region (180). Recent findings suggest that the DM15 region plays a role in specialized cap binding of TOP mRNAs (181), which may impede access of eIF4E to the cap. LARP1 was also shown to interact with the poly(A) tail of TOP transcripts (179), but the region within LARP1 responsible for this interaction remains unknown.

The exact role of LARP1 in the regulation of TOP mRNAs remains controversial. While LARP1 was shown to regulate TOP mRNA stability, it has also been described as a positive or negative regulator of TOP mRNA translation, depending on the context. Current evidence indicate that LARP1 binding to TOP mRNAs may inhibit their translation in response to mTORC1 inhibitors (23, 178) (Table 1). LARP1 may thus serve as a phosphorylation-sensitive switch that regulates the translation of TOP mRNAs. This model was recently supported by *in vitro* evidence using cell-free translation systems (182). Complementary strategies involving cell type- and organ-specific conditional knockout of LARP1 in whole animals will also be required in the future. Together, these approaches should help determine whether LARP1 is the long-sought regulator of TOP mRNA translation downstream of mTORC1. A recent study has shown that LARP1 is lost in *5q*^−^ syndrome, which is a macrocytic anemia characterized by defects in ribosome biogenesis (183).

### Additional mTOR targets implicated in translational control.

Besides LARP1, 4E-BPs, and S6Ks, mTORC1 directly phosphorylates eIF4G at multiple residues (184), but the functional consequences remain unknown. mTORC1 also increases ribosome biogenesis and tRNA synthesis rates. This is mediated chiefly by TIF-IA (185) and the RNA polymerase III repressor Maf1, respectively (186–189) (Fig. 1). mTORC1 may also promote mRNA translation by suppressing a selective autophagic pathway for 60S ribosomal subunits (i.e., ribophagy) (190, 191), but the specificity of this response in mammalian cells compared to bulk autophagy remains poorly understood (192).

## SELECTIVE TRANSLATION REGULATION BY mTOR

mRNAs exhibit differential translation activity based on their intrinsic features, which can sometime determine the rate of ribosome attachment (e.g., for mRNAs coding for α- versus β-globin) (193). This allows selective induction of translation of a subset of mRNAs in response to a variety of extracellular stimuli and intracellular cues, which is achieved by modulating the activities of components of the translational machinery by signaling pathways (Table 2). New technologies, including ribosome and polysome profiling, enabled investigation of translation on a transcriptome-wide scale (194, 195). These analyses further corroborated the tenet that translation plays an evolutionarily conserved role in shaping the proteomes, in particular during acute responses to stimuli.

TOP mRNAs harbor an extreme 5-terminal oligopyrimidine tract (5′TOP) which is characterized by a cytosine immediately following the cap followed by 4 to 15 uninterrupted pyrimidines (reviewed in reference 112). TOP mRNAs almost exclusively encode components of the translational apparatus (reviewed in reference 112). Inhibition of mTOR signaling leads to a reduction in TOP mRNA translation due to impaired initiation. Based on the observation that TOP mRNA translation is suppressed upon amino acid withdrawal, which is paralleled by downregulation of S6K activity and rpS6 phosphorylation, it was proposed that the S6Ks/rpS6 axis promotes TOP mRNA translation (140, 196, 197). Subsequent studies, however, demonstrated that neither ablation of S6Ks nor alterations in rpS6 phosphorylation influence TOP mRNA translation (122, 148, 198). More recently, ribosome profiling studies suggested that TOP mRNA translation is regulated by 4E-BPs (199). This conclusion was based on the findings that mTOR inhibitors suppress translation of TOP mRNAs more strongly in wild-type than in 4E-BP1/2 knockout mouse embryonic fibroblasts (MEFs). This was in contrast to previous findings of relative insensitivity of TOP mRNA translation to changes in eIF4E availability (200). Moreover, physiological stimuli, such as oxygen, growth factors, or nutrients, alter TOP mRNA translation in an mTOR-dependent but 4E-BP-independent manner (201). Hence, it appears likely that mTOR-mediated regulation of TOP mRNAs includes pathways that are independent from the 4E-BPs.

In addition to LARP1, which was described above, T-cell intracellular antigen 1 (TIA-1) and TIA-1-related protein (TIAR) were shown to suppress TOP mRNA translation specifically upon amino acid withdrawal (202) (Fig. 2). TIA-1 and TIAR are mRNA-binding proteins that aggregate within stress granules (203), but the role of mTOR in TIA-1/TIAR-mediated repression of TOP mRNAs remains unclear. These findings highlight the need to further investigate the mechanisms by which mTOR mediates TOP mRNA translation. Intriguingly, although mTOR depletion abrogates the effects of physiological stimuli on TOP mRNA translation, this is not the case when raptor or rictor is depleted (204, 205), suggesting that an mTOR complex other than mTORC1 or mTORC2 may be implicated in regulation of TOP mRNA translation.

Ribosome profiling studies suggested that mTOR almost exclusively regulates translation of mRNAs that harbor TOP or TOP-like motifs (199, 206), although the significance of the latter motifs has been questioned (207). Comparative analyses of several data sets derived from such analyses revealed the loss of representation of a large majority of bona fide non-TOP mRNAs (112). Indeed, polysome profiling studies suggested that, in addition to TOP mRNAs, alterations in mTOR activity impact non-TOP mRNAs (208).

Non-TOP mRNAs include a large proportion of transcripts whose translation is highly dependent on changes in 4E-BP activity and/or eIF4E levels (209, 210). Changes in 4E-BPs levels and/or phosphorylation dramatically alter translation of a subset of mRNAs (e.g., *IRF7*, *GAS2*, *CCND3*, *ODC1*, and *VEGFA*), while having relatively modest effects on global protein synthesis (120, 211–213) (Fig. 2). This pool of mRNAs largely overlaps those whose translation activity is dramatically affected by alterations in eIF4E levels (4, 209, 210, 214). These “eIF4E-sensitive mRNAs” are thought to harbor long and structured 5′ untranslated regions (5′UTRs) that render them critically dependent on the unwinding activity of the eIF4A subunit of the eIF4F complex (209, 215–217). The ability of eIF4A to efficiently unwind secondary RNA structures is strongly induced in the eIF4F complex (218, 219), and thus it is thought that recruitment of eIF4A to the eIF4F complex by eIF4E underpins the observed “eIF4E sensitivity” (reviewed in references 9 and 4). In contrast, mRNAs with 5′UTRs that are of the optimal length (70 to 150 nucleotides [nt] in mammalian cells) (220), including those encoding housekeeping proteins (e.g., actins and tubulins), exhibit minimal sensitivity to eIF4E (reviewed in reference 210).

The discrepancy between the ribosome and polysome profiling studies can be explained by the differences in the technology biases and experimental conditions that were used (221). NanoCAGE technology, which allows determination of transcription start sites on a genome-wide scale, confirmed that a large number of non-TOP mRNAs are mTOR sensitive (222). Among these non-TOP mRNAs, a subclass of transcripts with exceedingly short 5′UTRs (<50 nt) were also identified as being translationally regulated via the mTOR/4E-BP/eIF4E axis (Fig. 2). The vast majority of these mRNAs correspond to nuclear genes with mitochondrial functions, including components of the respiratory chain complexes (e.g., *ATP5O*, *ATP5D*, *UQCC2*, and *NDUF6*) (222). These transcripts frequently harbor a TISU (translation initiator of short 5′ UTR) element, and their translation is strongly affected by alterations in eIF4E but not eIF4A (222, 223). Mechanisms underlying the translation of TISU mRNAs are still not completely clear, but they may involve an interaction between eIF4G1 and eIF1, which facilitates dissociation of eIF4E from the 5′mRNA cap (224), followed by the eIF1A-directed association between the TISU element and RPS3 and RPS10e in the 48S complex and 80S monosome, respectively (225). Collectively, these findings suggest that mTOR regulates translation of mRNAs with specific 5′UTR features, including a number of non-TOP mRNAs.

mTORC1 can independently regulate each component of the eIF4F complex (eIF4E and eIF4A via modulating 4E-BP and PDCD4 phosphorylation, respectively, and eIF4G by direct phosphorylation). This suggests that the effects of mTOR on the translatome may be influenced by factors such as stoichiometry of the eIF4F components in the cell and/or the nature of the stimulus. Indeed, although changes in the levels and/or activity of the eIF4F complex subunits correspond to overlapping changes in the translatome, there are many notable differences. eIF4A and eIF4G affect different transcripts, and these are often distinct from those exhibiting eIF4E sensitivity (226–229). Moreover, mTOR regulates the phosphorylation of eIFs other than eIF4F components that selectively affect translation. For instance, eIF4B, which acts as an auxiliary factor that bolsters eIF4A helicase activity, is a substrate of S6Ks that has been demonstrated to selectively upregulate translation of mRNAs encoding survival and proliferation-promoting factors, including c-Myc, XIAP Cdc25, ODC, and Bcl-2 (230, 231) (Fig. 2). eIF4A was shown to possess oncogenic properties in T-cell acute lymphoblastic leukemia, as it was found to be particularly important for the translation of *MYC*, *NOTCH1*, and *MDM2* mRNAs (229). mTOR also seems to collaborate with CK2 in phosphorylating eIF2β (on Ser2 and Ser67 in human protein), which leads to translational inactivation of upstream open reading frame (uORF)-containing mRNAs (232). Finally, it has been suggested that mTORC1 can bolster translation of cyclin D3 mRNA by increasing its elongation rates via the eEF2K/eEF2 axis (158).

Altogether, these findings demonstrate that further studies are warranted to fully catalogue mTOR-sensitive mRNAs and identify the precise mechanism underpinning the qualitative and quantitative alterations of the translatome by mTOR.

## MAPK SIGNALING TO THE TRANSLATIONAL MACHINERY

The mitogen-activated protein kinases (MAPKs) are Ser/Thr kinases that regulate many essential processes, including gene expression, mitosis, metabolism, motility, survival, apoptosis, and differentiation (233). In mammalian cells, three MAPK families, i.e., ERK1/2, Jun N-terminal protein kinase (JNK), and p38 kinase, have been extensively characterized (reviewed in references 234, 235, 236, and 237). Each group of MAPKs function within a module composed of conserved, sequentially acting kinases: a MAPK, a MAPK kinase (MAPKK), and a MAPKK kinase (MAPKKK) (Fig. 3). The MAPK-activated protein kinases (MAPKAPKs), one of the many substrates of ERK1/2 and p38, are a family of Ser/Thr kinases which includes the p90 ribosomal S6 kinases (RSKs) and the MAPK-interacting kinases (MNKs) (238, 239). While multiple MAPKAPs have been shown to regulate gene expression, the RSKs and MNKs have been directly implicated in the regulation of mRNA translation (240, 241) (Fig. 3).

### MNKs.

In mammals, eIF4E is regulated by phosphorylation of a C-terminal residue (Ser209 in human eIF4E) (242, 243) by the MNKs (244, 245) (Fig. 3). There are two *MNK* genes in the human genome (*MKNK1* and *MKNK2*), each encoding two spliced isoforms (MNK1a and -b and MNK2a and -b) (reviewed in reference 240). While the longer forms, MNK1a and MNK2a, are primarily cytoplasmic, MNK1b and MNK2b are equally distributed between the nucleus and the cytoplasm (246, 247). Although both MNK1a and MNK2a contain a canonical MAPK-binding motif, their sequences differ slightly such that MNK1a binds both ERK1/2 and p38 kinases, while MNK2a associates only with ERK1/2 (245). The basal activity of MNK2a is high relative to that of MNK1a due to its sustained association with ERK1/2 (248). MNK1a has low basal activity but is responsive to the ERK1/2 and p38 activation (150, 248). Although MNK1b and MNK2b lack MAPK-binding motifs, these isoforms were shown to have high and low basal activity, respectively (247, 249). In mice, however, only MNK1a and MNK2a isoforms have been identified (reviewed in reference 240).

MNKs are recruited to eIF4E through association of their N-terminal regions with the C-terminal part of eIF4G (250). Phosphorylation of eIF4E is restricted to metazoans, as yeast lacks MNK orthologues. Although studies in Drosophila revealed that eIF4E phosphorylation is required for normal development (251), studies in MNK1/2 double knockout (DKO) mice or mice in which wild-type eIF4E was replaced with a nonphosphorylatable mutant (S209A) develop normally (252, 253). eIF4E phosphorylation, however, appears to be important in cancer (reviewed in reference 254). The nonphosphorylatable eIF4E mutant is less effective in transforming cells than the wild-type protein, *in vitro* and *in vivo* (255, 256). Similarly, mouse embryonic fibroblasts derived from MNK1/2 DKO animals were found to be resistant to Ras-mediated transformation (257), suggesting the importance of the MNK/eIF4E pathway in tumorigenesis.

MNKs are recruited to eIF4E via eIF4G (250), and thus, it is likely that eIF4E phosphorylation occurs during or after the eIF4F complex assembly. Ser209 is located near the cap-binding pocket of eIF4E, and its phosphorylation was initially predicted to stabilize eIF4E-cap interaction (258, 259). Subsequent studies revealed, however, that eIF4E phosphorylation reduces its cap affinity (260, 261), and depending on the experimental conditions, eIF4E phosphorylation was shown to correlate with increased (262–265) or decreased (266–268) global mRNA translation rates. Intriguingly, mRNAs which are sensitive to eIF4E level changes do not significantly overlap those whose translation is altered by changes in the eIF4E phosphorylation. Phosphorylated eIF4E appears to stimulate translation of mRNAs (e.g., *SNAI1*, *MMP3*, and *VIM*) encoding proteins involved in migration and metastasis and/or inflammation (cytokines) (252, 269, 270) (Fig. 2). This suggests that unlike eIF4E overexpression, which stimulates tumor initiation, eIF4E phosphorylation facilitates tumor progression by increasing metastatic potential via selective upregulation of translation of mRNAs encoding proteins critical for remodeling of the extracellular matrix, epithelial-to-mesenchymal transition, and inflammation. Given the roles of MNKs in tumorigenesis and the fact they are dispensable for animal growth and development (271), recently identified MNK1/2 inhibitors (eFT508 and BAY 1143269) (Table 1) are currently being tested in phase I/II clinical trials in patients suffering from hematological malignancies or solid tumors (272).

### RSKs.

The RSK family comprises four highly similar members (RSK1, RSK2, RSK3, and RSK4) that are activated by Ras/MAPK signaling (reviewed in references 241 and 273) (Fig. 3). ERK1/2 interact with a D-type docking motif in the RSK C-terminal region (274) and promote the phosphorylation of several Ser/Thr residues present in all RSK isoforms. RSK proteins exist in all vertebrate species, and related orthologues have been identified in Drosophila and Caenorhabditis elegans but not in yeast. With the exception of RSK4, all RSK are ubiquitously expressed in developing and adult tissues (275). RSK1, RSK2, and RSK3 are usually present in the cytoplasm but translocate into the nucleus in response to stimulation (276, 277). RSK4 does not significantly accumulate in the nucleus following stimulation of the Ras/MAPK pathway (278). An important feature of RSK is that it contains two distinct and functional kinase domains. The C-terminal kinase domain (CTKD), which belongs to the CAMK (Ca^2+^/calmodulin-dependent protein kinase), family is responsible for receiving an activating signal from ERK1/2 which is transmitted to the N-terminal kinase domain (NTKD) that phosphorylates substrates (279, 280). The NTKD belongs to the AGC family of protein kinases and targets basic phosphorylation motifs (164), explaining why many RSK substrates are shared with AKT and S6Ks (273).

Over 30 years ago, RSK was identified as an rpS6 kinase in unfertilized Xenopus laevis eggs, which suggested that it may regulate translation (281). S6K1 and S6K2 were later shown to be the predominant rpS6 kinases in somatic cells (282, 283). Subsequent studies using S6K1^−/−^ S6K2^−/−^ cells confirmed these findings but also showed residual rpS6 phosphorylation by RSK (122). Both RSK1 and RSK2 were found to specifically phosphorylate rpS6 on Ser235 and Ser236 (147). The functional role of rpS6 phosphorylation is largely unknown (148, 284); however, these results suggest that RSK provides an mTOR/S6K-independent input linking MAPK signaling to the potential regulation of mRNA translation.

Ras/MAPK signaling impinges on the PI3K/mTOR pathway at various levels to regulate translation. In addition, RSK directly regulates components of the translation apparatus (Table 2), such as rpS6 (Ser235/236 in humans) (147), eIF4B (Ser422 in humans) (171), and eEF2K (Ser366 in humans) (150) (Fig. 3). Phosphorylation of eIF4B promotes its interaction with eIF3, which correlates with increased translation rates (171, 173). RSKs and S6Ks regulate eIF4B phosphorylation with different kinetics, which may explain the biphasic pattern of eIF4B phosphorylation observed in response to growth factors (171). RSKs stimulate PDCD4 phosphorylation and degradation (164) and phosphorylate eEF2K at Ser366 (150), which leads to its inhibition. As was shown with AKT and S6K (285), RSK-mediated phosphorylation and inhibition of GSK3β (on Ser9 in humans) (286) may activate eIF2B, a key regulator of protein synthesis (287). In collaboration with ERK1/2, RSKs was suggested to contribute to rRNA synthesis by phosphorylating TIF-1A (Ser633 and Ser649 in humans, respectively), but these sites do not appear to lie within RSK consensus phosphorylation sequences (288). Nonetheless, these phosphorylation events were shown to be dependent on the Ras/MAPK pathway. Together these data demonstrate that RSKs play a major role in the regulation of mRNA translation (Fig. 3).

## REGULATION OF TRANSLATIONAL MACHINERY BY PHOSPHATASES

In contrast to the extensive literature on the role of protein kinases in the regulation of translation, the role of protein phosphatases in protein synthesis remains largely underexplored. As described elsewhere (9, 10), serine/threonine phosphatase complexes containing PPP1R15 family member GADD34 or CReP, in conjunction with protein phosphatase 1C (PP1C), play a major role in regulation of eIF2α phosphorylation and thus ternary complex recycling (289, 290). In addition, phosphatases other than lipid phosphatase PTEN have been shown to regulate mTOR signaling and thus mRNA translation. For instance, TORC1 inhibition in S. cerevisiae leads to dissociation of the serine/threonine phosphatase SIT4 from its inhibitor TAP42, which results in dephosphorylation and activation of the eIF2α kinase GCN2 (291). In mammals, inhibition of mTORC1 activates the SIT4 orthologue PP6C, which stimulates GCN2 and induces eIF2α phosphorylation (292).

A number of serine/threonine phosphatases have also been proposed to act upstream of mTOR. For example, protein phosphatase 2A (PP2A) inactivates AKT by dephosphorylating its active site (Thr308 in humans) (293). In turn, the PH domain leucine-rich repeat protein phosphatases (PHLPP1 and PHLPP2) dephosphorylate AKT on its hydrophobic motif (Ser473 in humans) (294), whereby the loss of PHLPP activity, which appears to frequently occur in cancer, results in AKT hyperphosphorylation (295). Mechanisms whereby phosphatases act downstream of mTOR have also been proposed. It was suggested that mTOR controls phosphorylation of S6Ks and 4E-BPs by suppressing their dephosphorylation by PP2A. PP2A was shown to associate with S6Ks and be activated by rapamycin (296). It also appears that 4E-BP1 may be dephosphorylated by the serine/threonine phosphatase PPM1G, which in glioblastoma cell lines leads to translational upregulation of the helix-loop-helix transcriptional modulator Id1 (297, 298). Moreover, attachment of lung fibroblasts to the collagen matrix has been reported to activate the β1 integrin/Src/PP2A axis, leading to 4E-BP1 degradation and increased cap-dependent translation (299). In contrast, PP2A-dependent dephosphorylation of 4E-BP1 has been proposed to underpin PKCα-mediated suppression of cap-dependent translation and proliferation of intestinal epithelial cells (300). Finally, inhibition of protein synthesis by 2-deoxyglucose (2-DG), which acts as a potent inhibitor of glycolysis, is thought to be at least in part mediated by dephosphorylation of 4E-BP1 by PP1/PP2A and PPM1 phosphatases (297, 301).

In addition to 4E-BPs and S6Ks, other downstream effectors of mTORC1 implicated in regulation of protein synthesis have been shown to be controlled by phosphatases. For example, insulin regulates eEF2 dephosphorylation via PP2A (302). Phosphatases are also thought to regulate eIF4E phosphorylation levels. PP2A, for example, has been shown to decrease eIF4E phosphorylation at Ser209, which occurs both directly and indirectly via suppression of MNKs. Decreased phosphorylation subsequently impedes assembly of the eIF4F complex (303). In turn, it has been suggested that translation of immunoglobulin-binding protein 1 (Igbp1), which is a regulatory subunit of PP2A, is eIF4E sensitive (304). Finally, ribosomal proteins, including rpL5 and rpS6, have also been shown to be targeted by PP1 (305, 306). Collectively, these findings strongly suggest an important role for phosphatases in the regulation of translation, but future studies are required to establish the precise molecular mechanisms underpinning the action of phosphatases toward the translational apparatus, as well as the biological significance of these phenomena.

## CONCLUDING REMARKS

While significant progress has been made toward understanding how signaling pathways, such as PI3K/mTOR and Ras/MAPK, regulate the phosphorylation of components of the translation apparatus, very little is known about how these events regulate mRNA translation or the translatome. This is particularly important, as these signaling pathways are comprised of several oncogenes and tumor suppressors, which are often dysregulated in cancer (58). Notwithstanding the relatively comprehensive understanding of mTOR signaling to the translational apparatus (221, 307), outstanding questions regarding the mechanisms of selective modulation of the translatome by mTOR still remain (112). Perhaps the biggest riddle of all is the TOP mRNAs, whose regulation remains poorly understood despite decades of work since their discovery as rapamycin-sensitive transcripts (308). Notwithstanding the large body of work surrounding the regulation of mRNAs with structured 5′UTRs or with extremely short 5′UTRs (reviewed in reference 221), several questions remain about the mechanisms by which mTOR regulates their translation. Recent efforts to catalogue mTOR-sensitive transcripts using ribosome or polysome profiling resulted in conflicting results that are likely explained by analytical and technical biases (222). Moreover, the lack of reliable UTR databases reduces the accuracy of experimental findings. Future efforts will have to consider all these points to improve study design and data analysis. Recent advances in pharmacological tools (e.g., compounds that specifically inhibit mTOR or eIF4F components), genetic tools (e.g., clustered regularly interspaced short palindromic repeat [CRISPR]/Cas9-based genome manipulations), and technologies that enable genome-wide monitoring of changes in the translatome (e.g., ribosome profiling/transcriptome sequencing [RNA-seq]) or the proteome (e.g., quantitative mass spectrometry) will undoubtedly help decipher the role of signaling pathways in translation regulation as it relates to homeostasis and disease.

While mTOR and MAPK pathways, as well as eIF2α kinases, play pivotal roles in the regulation of protein synthesis, several additional signaling pathways have been implicated in the phosphorylation of components of the translational machinery and auxiliary factors (e.g., PAK2, GSK3, Cdk11, CK1, PKC, and CK2) (126, 127, 232, 309–311). Notwithstanding the fact that these protein kinases specifically regulate components of the translation machinery, their physiological role in translational control remains obscure. Together with the differential expression of ribosomal proteins and rRNA, these phosphorylation events may participate in the generation of specialized ribosomes, which would have a substantial impact on how mRNAs are translated into functional proteins (reviewed in reference 312). Related to this is the recent demonstration that rpS6 phosphorylation has a broad influence on the transcription of genes involved in the ribosome biogenesis (RiBi) program (149), suggesting that posttranslational modification of a ribosomal protein may facilitate the synthesis of RiBi factors. While many questions remain, the next few years are anticipated to bring new and exciting discoveries on the role of signaling pathways in global and specific mRNA translation. These results are likely to improve understanding of the etiopathology of many human disorders and diseases that are linked to defective translational control, such as cancer (34, 206), neurological disorders (313, 314), and aging (315, 316).
